# Comparing interfacial dynamics in protein-protein complexes: an elastic network approach

**DOI:** 10.1186/1472-6807-10-26

**Published:** 2010-08-08

**Authors:** Andrea Zen, Cristian Micheletti, Ozlem Keskin, Ruth Nussinov

**Affiliations:** 1 SISSA, Democritos CNR-IOM and Italian Institute of Technology, Via Bonomea 265, 34136 Trieste, Italy; 2 Center for Computational Biology and Bioinformatics and College of Engineering, Koc University, Rumelifeneri Yolu, 34450 Sariyer Istanbul, Turkey; 3 Basic Science Program, SAIC-Frederick, Inc., Center for Cancer Research Nanobiology Program, NCI-Frederick, Frederick, MD 21702, USA; 4 Sackler Institute of Molecular Medicine, Department of Human Genetics and Molecular Medicine, Sackler School of Medicine, Tel Aviv University, Tel Aviv 69978, Israel

## Abstract

**Background:**

The transient, or permanent, association of proteins to form organized complexes is one of the most common mechanisms of regulation of biological processes. Systematic physico-chemical studies of the binding interfaces have previously shown that a key mechanism for the formation/stabilization of dimers is the steric and chemical complementarity of the two semi-interfaces. The role of the fluctuation dynamics at the interface of the interacting subunits, although expectedly important, proved more elusive to characterize. The aim of the present computational study is to gain insight into salient dynamics-based aspects of protein-protein interfaces.

**Results:**

The interface dynamics was characterized by means of an elastic network model for 22 representative dimers covering three main interface types. The three groups gather dimers sharing the same interface but with good (type I) or poor (type II) similarity of the overall fold, or dimers sharing only one of the semi-interfaces (type III). The set comprises obligate dimers, which are complexes for which no structural representative of the free form(s) is available. Considerations were accordingly limited to bound and unbound forms of the monomeric subunits of the dimers. We proceeded by first computing the mobility of amino acids at the interface of the bound forms and compare it with the mobility of (i) other surface amino acids (ii) interface amino acids in the unbound forms. In both cases different dynamic patterns were observed across interface types and depending on whether the interface belongs to an obligate or non-obligate complex.

**Conclusions:**

The comparative investigation indicated that the mobility of amino acids at the dimeric interface is generally lower than for other amino acids at the protein surface. The change in interfacial mobility upon removing "in silico" the partner monomer (unbound form) was next found to be correlated with the interface type, size and obligate nature of the complex. In particular, going from the unbound to the bound forms, the interfacial mobility is noticeably reduced for dimers with type I interfaces, while it is largely unchanged for type II ones. The results suggest that these structurally- and biologically-different types of interfaces are stabilized by different balancing mechanisms between enthalpy and conformational entropy.

## Background

Since key biological processes such as antigen-antibody recognition, hormone-receptor binding and signal transduction are regulated through association and dissociation of proteins, characterizing the physico-chemical properties of protein-protein interactions has always been a primary aim of molecular biology. Several studies have systematically addressed protein-protein interactions and their applications ranging from rational drug design to structure prediction of multimeric complexes [1-10]. Among these [1,11-15] some focused on the complementarity of chemical and structural features (shape, hydrophobic patterns, distribution of electrical charge) at the binding interface as major contributors to intersubunit interactions. Typically, these can be well characterized by using a single reference configuration for the interacting subunits, that is, by treating them as if they were rigid molecules. In recent years, thanks to the increasing availability of computational resources and more refined theoretical models, it has been possible to systematically investigate the role of a complementary physical effect, namely the internal dynamics of the interacting subunits. A number of studies [16-19] have shown that even in the absence of the partner subunits, the dynamical properties of amino acids at the known interface region can differ from those of other amino acids at the protein surface. Although the results have been established in a limited number of contexts they may point to a general mechanism, namely that protein regions that are capable of binding a specific partner could present not only specific shape and chemical composition but also a specific fluctuation dynamics in the *free *form.

This effect would represent a non-trivial generalization of the mechanism recently proposed by Aden *et al*. [20] according to which the internal dynamics of certain enzymes is innately predisposed to assist, even in the absence of the substrate to be processed, the conformational changes required by the enzymatic catalysis, such as the formation of a transition-state structure [21]. In several single-molecule experiments and in computational studies various enzymes in the apo form have been shown to be capable of interconverting between the rest and catalytically-competent conformations [22-30]. This view presents an alternative to the induced-fit mechanism, where it is the tight interaction of the partners that profoundly modifies the free-energy profiles thus triggering the conformational change between the competing biologically-relevant conformations.

Recent studies have shown that comparisons of salient aspects of the internal dynamics in different proteins can be profitably used to pinpoint key common aspects (usually functionally-oriented) that could be more elusive to structure-based analysis [31-34]. It appears natural to carry out a similar analysis also in the context of protein-protein complexes. In particular, it is envisaged that a systematic investigation and comparison of the salient dynamical properties across different types of protein assemblies could highlight traits that depend on fundamental properties of the complexed monomers or their binding interface. An ideal context for undertaking such investigation is offered by the database of representative protein interfaces recently assembled by Keskin *et al*. [35,36]. This study considered all the interfaces between pairs of protein chains in available PDB entries. After removal of sequence redundancy all complexes with structurally-similar interfaces were grouped in the same cluster. Each cluster was assigned to one of three main types of interfaces [35,37,38]. Type I gathers clusters whose members are structurally similar not only at the interface, but over the entire interacting units. Type II includes clusters whose members are only similar at the interface region (both sides); the members of these groups have a different overall structural organization. Finally, type III groups are characterized by the fact that their members share only one side of the interface region (i.e. a semi-interface). It was observed that parent proteins of members of the same type I cluster belong to the same functional family, while parent proteins of members of the same type II or III cluster may belong to different functional families [35,37,38]. The set contained both obligate and non-obligate complexes. In the former case the monomers taking part to the complex are not stable on their own; in the latter case they are stable also in the free form.

In the present computational/theoretical investigation, the above-mentioned classification scheme is used to understand whether the three main types of interfaces are characterized by different properties of their fluctuation dynamics in thermal equilibrium. The latter is computed by means of a phenomenological elastic network model (ENM) [39-52]. ENMs are known to be capable of identifying reliably, at a negligible computational cost, the collective low-energy modes controlling the structural fluctuations around a given protein conformation. Therefore they are well suited to the present context where 22 complexes are considered. The large number of instances make, in fact, impractical the use of other approaches based on atomistic interaction potentials such as molecular dynamics.

In our ENM analysis we have evaluated the mobility of the amino acids in the proteins of our dataset, particularly of those taking part to the dimeric interfaces. The mobility of the semi-interfaces is examined both in the *bound *form (i.e. considering the presence of the partner monomer) and in the *unbound *one, corresponding to the structurally-quenched monomer resulting from the "*in silico*" splitting of the complex. For non-obligate interfaces, the unbound form is generally a viable structural proxy for the *free *form, while this is not true for obligate-complexes due to the instability of the individual monomers. In consideration of this fact, the comparison of the properties of the bound and unbound forms cannot be used to shed light on the process of dimer formation. It is used, instead, to understand the extent to which the mobility at a dimeric interface reflects the intrinsic, innate, mobility of the two semi-interfaces, or results from their mutual structural-dynamical interplay.

The first element provided by our investigation is that the overall mobility at the bound semi-interface is lower than other surface regions. This fact is in accord with previous findings [16-19]. The comparison of bound and unbound forms further indicated that the degree to which the mobility of amino acids at a dimer semi-interface is affected by the bound partner monomer depends on the interface type. Specifically, significant bound/unbound mobility differences are seen for obligate, large and/or structurally-specific interfaces, while non-appreciable differences are found for non-obligate, medium-sized and/or unspecific interfaces.

The results suggest that, depending on the interface types, partner semi-interfaces have a different impact on restraining and shaping the dimeric interfacial mobility. This, in turn, should reflect a different interplay of enthalpy and conformational entropy in the increase of free energy upon separation of the structurally-quenched monomers.

## Results and Discussion

Hereafter we shall provide a detailed characterization of the equilibrium fluctuation dynamics of a representative set of dimeric protein complexes. The section has three parts. The first illustrates the criteria used to select the representative protein-protein complexes. In the second part the salient structural traits of the complexes and their interfacial regions are discussed. Finally we conclude with a detailed discussion of the fluctuation dynamics of the interfaces focusing on the differences across the three main classes.

### Selection of protein-protein complexes

The dataset, covering diverse dimeric interfaces was created starting from the comprehensive list of known protein-protein interfaces [35,36]. This set was obtained by (i) parsing the complete set of pairs of contacting protein chains contained in all PDB entries, (ii) selecting the pairs of chains with a sizable binding region and (iii) clustering their interfaces on the basis of structural similarity. Each cluster was assigned to one of three main types of interfaces described in the introduction.

For the purpose of the present study, we need to select a set of representatives of the various interface clusters. The comprehensive dataset of Keskin *et al*. was culled on the basis of two criteria. First, considerations are restricted to dimeric protein interfaces. Next, we considered only dimers whose internal fluctuation dynamics is expected to be adequately captured by elastic network models (see Methods section for details on the filtering procedure). Because these computationally-effective models are appropriate for characterizing the equilibrium fluctuation dynamics of compact globular proteins, we restricted our considerations to dimers without long exposed loops or termini as their diffusive motion would not be adequately captured by the elastic network approach (described in the Methods section). Finally, to limit the structural redundancy, each cluster was represented by a single entry, the first one provided by the study of ref. [35].

The application of these selection criteria resulted in the dataset of 22 dimers reported in Table 1. Eight dimers have an interface classified as type I, six have an interface of type II and eight have a type III interface.

**Table 1:** List of protein-protein interfaces investigated

				Chain Size (a.a.)			Interface		**Semi-int**.		NOXclass interface
PDB	Chains		Classification	**1**^***st***^	**2**^***nd***^	both	**Å**^**2**^	**a.a**.	**1**^***st***^	**2**^***nd***^	
*Interfaces of Type I*											
1a03	A	B	calcium-binding protein	90	90	l80	307l	82	37	45	obligate
1gdh	A	B	oxidoreductase	320	320	640	6254	l48	74	74	obligate
1a05	A	B	oxidoreductase	357	357	7l4	5258	l25	62	63	obligate
1ag1	O	T	isomerase	249	249	498	3025	75	37	38	obligate
1hii	A	B	hydrolase	99	99	l98	3436	83	4l	42	obligate
1mdi	A	B	electron transport/peptide	l05	l3	ll8	l340	34	24	l0	non-obligate
2gsa	A	B	chlorophyll biosynthesis	427	427	854	9l78	233	ll8	ll5	obligate
1ger	A	B	oxidoreductase	448	448	896	6777	l72	86	86	obligate
											
*Interfaces ofType II*											
1azy	A	B	glicosyltransferase	440	440	880	l744	42	2l	2l	non-biological
1a0a	A	B	transcription factor	63	63	l26	l860	44	22	22	obligate
1mv4	A	B	de novo protein	37	37	74	l833	43	2l	22	obligate
1a93	A	B	leucine zipper	32	32	64	l565	34	l7	l7	non-obligate
1a15	A	B	chemokine	67	67	l24	l480	38	l8	20	obligate
1q6a	A	B	circadian clock protein	l07	l07	2l4	l8l0	46	23	23	non-obligate
											
*Interfaces ofType III*											
2fhw	B	A	signaling protein	27	24	5l	ll53	29	l5	l4	obligate
1jm7	A	B	antitumor	l03	97	200	2769	72	39	33	obligate
1tmz	A	B	tropomyosin	32	32	64	l586	35	l7	l8	obligate
1an2	A	C	DNA-binding protein	86	86	l72	257l	64	32	32	obligate
2sic	E	I	proteinase/inhibitor	275	l07	382	l6l7	45	3l	l4	non-obligate
1lfa	A	B	cell adhesion	l83	l83	365	628	2l	l0	ll	non-biological
1shc	A	B	signal transduction/peptide	l95	ll	206	l456	34	25	9	non-obligate
2a93	A	B	leucine zipper	32	32	64	l568	34	l6	l8	non-obligate

For each protein complex investigated in this work it is given: the PDB ID; the chains that form the considered interface; the classification, according to PRISM http://prism.ccbb.ku.edu.tr/prism/; the number of amino acids for the first and second chain listed, and the total number; the size of the interface in Å^2 ^and in number of amino acids involved; the number of interface amino acids in the first and second chain; the classification of the interface into biological obligate, biological non-obligate or non-biological, according to NOXclass [68].

This classification scheme is complemented by consideration of whether a given protein-protein complex has a genuine biological origin or if it is the artifactual result of crystal packing in the X-ray resolved structure. The obligatory or non-obligatory character of the biological complexes was further considered, see Table 1. Overall there are: fourteen obligate interfaces (seven of type I, three of type II and four of type III); six non-obligate interfaces (one of type I, two of type II and three of type III); and two non-biological interfaces (one of type II and one of type III). Most dimers in Table 1 are, therefore, obligate complexes. In general, the subunits of a dimer have different structures in the bound and in free forms. For non-obligate interfaces, the bound-free conformational rearrangements are typically modest and are mostly localized at the interface region [2,15,17,30]. For obligate complexes, however, the changes are expected to be dramatic [53-55]. Obligate complexes often involve intrinsically disordered proteins, which are folded in the presence of the binding partner, but do not have a unique three dimensional structure in the isolated, free state. As anticipated in the Introduction, to treat on an equal footing the cases of obligate and non-obligate interfaces we shall base our considerations exclusively on the bound and unbound monomeric forms.

### Structural properties of dimers and their interfaces

Before considering the dynamical properties of the representative dimers, we discuss several (mostly structural) aspects.

**Figure 1:**
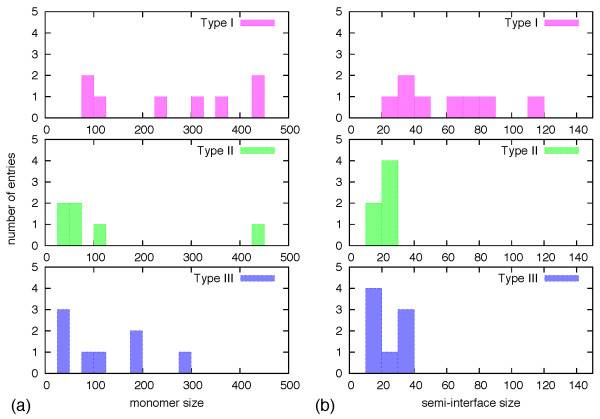
**Distribution of sizes of (a) the first chain of the dimers reported in Table 1, and of (b) their semi-interface region**.

(i) Protein length: As readily seen in Table 1, the dataset covers a wide range of lengths, from 64 [PDB:1a93, 1tmz, 2a93] to 896 amino acids for oxidoreductase [PDB:1ger]. Most of the largest complexes have type I interfaces. Five of the seven complexes which have monomers comprising more than 200 residues are of type I, one is of type II and one of type III. This property is readily perceived in Figure 1a, which provides a histogram of the sizes of the first chain in Table 1 for each complex.

(ii) Interface area: The interface area was computed following the standard procedures described in the Methods section. Similar to the case of dimers length, the interface lengths span a wide range of values (Table 1): from 21 amino acids (interface area: 628Å^2^) of the cell adhesion protein [PDB:1lfa] to 233 amino acids (interface area: 9178Å^2^) of chlorophyll biosynthesis [PDB:2gsa]. The largest interfaces are of type I. Seven of the eight interfaces of type I are larger than 3000Å^2^. By comparison, interfaces of type II and III are appreciably smaller, with areas in the 1400Å^2 ^- 1900Å^2 ^range, which is typical for medium size interfaces [2].

Figure 1b illustrates the size distribution of the semi-interface of the first chain for each dimer in Table 1. We observe that most of the semi-interfaces of type I consist of more than 30 amino acids, while those of types II and III involve less than 30 amino acids (we recall that members of type III groups share only one of the two semi-interfaces).

**Figure 2:**
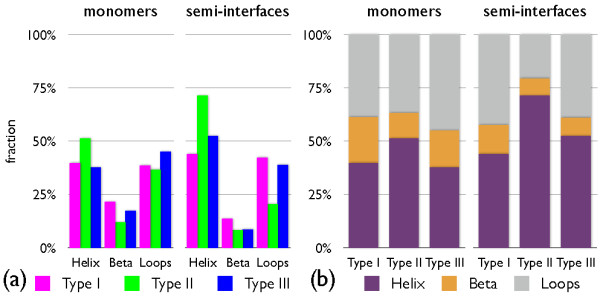
**Graphical summary of the fraction of secondary elements in the considered monomers and their semi-interfaces**. Data are presented separately for each of the three types of interfaces.

Inspection of Table 1 indicates that the largest interfaces pertain to large complexes. While this may be expected, for medium-sized interfaces there is no simple correlation between dimer size and interface size. For instance the leucine zipper [PDB:1a93], which is a dimer of 64 amino acids, has an interface of 1565Å^2^, which is larger than the interface of chemokine [PDB:1a15], a complex of 124 amino acids.

(iii) Secondary structure: The relative secondary content, subdivided according to the three types of interfaces are reported in figure 2. More than 50% of the amino acids of dimers with type II interfaces belong to helical secondary motifs, while the percentage decreases slightly to 40% for type I and III cases. The fraction of amino acids in coils is close to 40% for the complexes of the three types, and the fraction of amino acids in strands is smaller than 20%. In the semi-interfaces, there is an increase of helical content; more than 70% of the amino acids are in helical conformation for type II entries, while for type I and III the percentage is about 50%, with a related decrease in coil and strand content. Despite the reduced size of the representative set of dimeric entries, the overall secondary structure-content is compatible with the one found over the entire dataset of dimers considered in ref. [38].

### Dynamical properties of the dimers and their interfaces

The overall fluctuation dynamics of the amino acids at the dimeric interfaces was investigated with a twofold aim. First, the mobility of amino acids at the dimeric interface was compared with the mobility of other amino acids at the protein surface. The dynamical properties of amino acids at the known dimeric interfaces were next analyzed to ascertain which salient features (such as secondary and tertiary organization etc.) impact the fluctuation dynamics of the interface region.

#### Mobility of the interfacial amino acids

The mobility of the amino acids was studied using an elastic network model [39-52] which relies on a quadratic approximation for the near-native free energy landscape. Specifically, suitable quadratic (harmonic) penalties are used for the displacement of amino acids from their positions in the reference crystallographic structure. The quadratic approximation of the free energy has been shown to be adequate for capturing the salient features of the internal dynamics of globular proteins [56,57]. The specific elastic network employed here (see Methods) is the *β*-Gaussian network model [47] which adopts a two-centroid description for amino acid mobility (one centroid for the main-chain and one for the amino acid sidechain) and that has been previously validated, for several globular proteins, against extensive molecular dynamics simulations [47,58-60].

It is important to point out that the characterization of the mobility of amino acids in a protein implicitly depends on the prior subtraction of the underlying "rigid-body" motion (rotations and translations) of the molecular system. Almost invariably, the elimination of the rigid-body motion is carried out by minimizing the average mean-square displacement of *all *amino acids. In such a case, the center of mass of the entire protein (complex) of interest remains fixed in space. For multimeric or multidomain proteins this choice is not necessarily appropriate, as an appreciable relative motion of the protein dynamical domains [61,62] can lead to artifactual results [23].

In the present context, where protein dimers are considered, there are two possible natural choices for how to remove the rigid-body motion, according to whether one is interested in characterizing the fluctuation dynamics of the entire complex, or of only one subunit (whose dynamics is nevertheless affected by the presence of the partner subunit). The former choice corresponds to the standard procedure of minimizing the mean-square displacements of the *entire *complex. In the second case the minimization is carried out over only *one of the monomeric subunits*.

The latter choice will be adopted hereafter (unless otherwise specified) as it practically amounts to following the fluctuation dynamics of the subunit of interest in its "relative reference frame" through the removal of the overall rotations and translations of the C_*α *_trace of the subunit (and not of the entire system). The scheme is appropriate in view of the comparisons that will be carried out between the bound/unbound forms of the dimers. For subunits in the bound state, the amino acid mobility will be calculated by taking into account, through a thermodynamic averaging procedure (see Methods), the interaction with the partner monomer.

Because of the symmetry of the interfaces of type I and II dimers, the choice of which of the two subunit is considered is immaterial. This is not the case for dimers with type III interfaces, where only one side of the interface, by convention the one belonging to the first chain reported in Table 1, is shared by members of the same cluster. We will therefore systematically use the first chain reported in Table 1 to define the "subunit frame of reference" for all our dimers.

**Figure 3:**
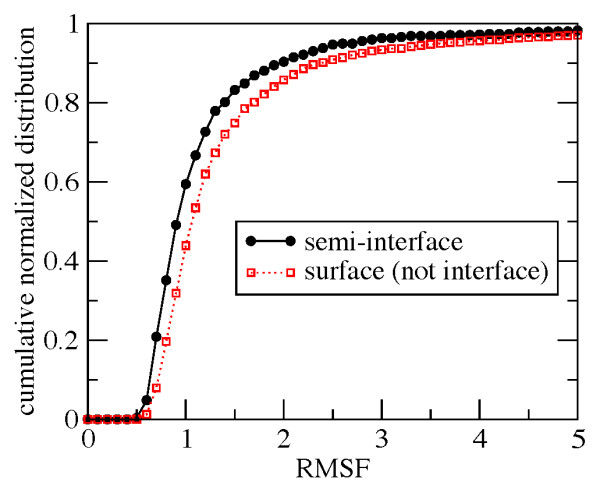
**Normalized cumulative distribution of the RMSF, in the units of the elastic network model, for residues at monomeric semi-interfaces, and non-interfacial surface regions**. The data are combined over all representative dimers.

We first compared the mobility of residues in the bound semi-interface of interest against the mobility of other surface residues. Considering all proteins in our dataset, the total number of residues in the monomeric units is 3774. According to standard criteria based on solvent accessibility (see methods), ~21% of such amino acids are at the semi-interface while ~57% of them are surface but not interface residues. Considering the solvent-exposed surface of the unbound forms most of the residues at the semi-interface (~96%) would be classified as surface amino acids. The normalized distribution of the root mean square fluctuations (RMSF) of residues at the semi-interface and that of surface residues which are not at the semi-interfaces are shown in Figure 3. In the units of the elastic network model, the average RMSF values of the two distributions are 1.19 and 1.54, respectively, while the distributions spreads (standard deviation) are 0.95 and 2.17, respectively. Consistently with intuitive expectations, the data indicate that residues at a semi-interface are, *on average*, less mobile than residues in non-interfacial surface regions. More quantitatively, the fraction of residues with RMSF lower than 1, a value indicative of an intermediate mobility, is ~60% for semi-interface residues, and ~44% for surface (but not interface) residues.

Considering the RMSF distribution separately for dimers with interface types I, II and III (see Table 1 and Figures 1, 2, 3 of the Additional file 1) interface residues are, on average, appreciably less mobile than surface residues for types I and III entries, but not for type II ones. The distribution of the RMSF is sharper for obligate complexes than for non-obligate ones (see Additional file 1).

#### Factors affecting the mobility of interfacial amino acids

A *priori*, the reduced mobility of amino acids at the bound semi-interfaces compared to other surface regions, could be ascribed to two main factors: (i) the intrinsic structural architecture of the individual monomeric unit (such as the locality of the inter-residue contacts) and/or (ii) the contact interactions with the partner monomer, which acts as a mobility-limiting constraint for the semi-interface.

The interplay of these two factors was considered earlier with regard to the mobility of free and bound forms of monomeric units [16,17,19]. As noted above, the bound form can be appropriately taken as a proxy for the free form for non-obligate interfaces. Several of the interfaces considered here are, however, obligate, implying that a well-structured free form of the monomer may not necessarily exist. Nonetheless, even without reference to the free form, several key aspects of the interplay between (i) and (ii) can be elucidated based on considerations of only bound and unbound forms.

Considerable insight can be gained by using an elastic network model to study the fluctuation dynamics of the monomer of interest and comparing the behavior when the partner monomer is present and when it is absent. If a realistic force-field were employed to study the dynamics of obligate dimers, the removal of the partner monomer would lead to a loss of structural organization of the subunit of interest; this is because the isolated monomer would not correspond to a minimum of the free energy. Instead, by resorting to an elastic network model (ENM), it is possible to study the "intrinsic" fluctuation dynamics of the subunit of interest because the ENM approach amounts to introducing a model free energy that, by construction, has a minimum in the input reference, unbound, structure.

In brief, the relative role of factors (i) and (ii) can be ascertained by an ENM calculation of the fluctuation dynamics of the monomer of interest either in the absence of the partner monomer or in its presence. In the latter case, a suitable thermodynamic integration of the degrees of freedom of the partner monomer needs to be carried out (see Methods). In can be anticipated that three possible scenarios can emerge from the comparison: (A) the fluctuations of the semi-interface residues are small both in the bound and in the unbound forms; (B) the fluctuations of the semi-interface residues are small in the bound form and large in the unbound one; (C) the fluctuations of the semi-interface residues are large both for the bound and the unbound forms. Since the partner monomer acts as a constraint for the mobility of the monomeric semi-interface, the fourth case, where the semi-interface is more mobile in the bound than the unbound form cannot occur.

Case (A) would indicate that factor (i), i.e. the structural architecture of the monomer, has the dominant influence on the mobility (the low degree of mobility, in this case) of the residues. In contrast, in case (B) the main player would be factor (ii), i.e. the constraints due to the interaction with the partner monomer. Case (C) is subtler as it would indicate that neither factors (i) and (ii) are responsible for the observed diminished mobility of the semi-interface residues, compared to other surface amino acids. It would be particularly interesting to observe that semi-interface fluctuations in the bound and unbound forms were similar, as this would indicate that the binding partner has an interface organized in a way that modestly interferes with the "innate", intrinsic, fluctuation dynamics of the monomer of interest.

**Figure 4:**
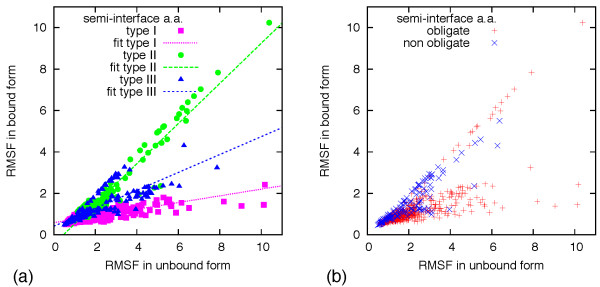
**Scatter plot of the mobility of amino acid at semi-interfaces of bound and unbound monomers**. (a) Interfaces of type I, II and III have been represented respectively with pink squares, green circles and blue triangles. Interpolating lines for each type of interface are also shown. (b) Interface residues for obligate and non-obligate complexes are represented respectively with red + and blue ×.

The scatter plots in Figure 4 illustrate the changes in mobility going from the unbound (*x *axis) to the bound form (*y *axis) for the residues at the first semi-interface of the studied complexes. Residues of interfaces of type I, II and III are respectively colored in pink, green and blue in panel (a) of Figure 4. In panel b of the same figure, residues from obligate and non-obligate complexes are respectively colored in red and blue. In these graphs, amino acids of dimers for which scenario (A) is applicable would appear in proximity of the origin. For scenario (B) the entries would mostly distribute along the *x *axis while for scenario (C) they would occupy the region of space *y *<*x*. In the notable case where the partner does not inuence the intrinsic uctuations of the first monomer, the entries would be distributed along the line *y *= *x*.

Figure 4 indicates that the three possible scenarios are all present, albeit with different weight. In fact, the first observation is that factors (i) and (ii) do not have systematically the same relative weight across the considered representatives. By inspecting these scatter plots for each interface separately (see Additional file 1), it emerges that the best examples of semi-interface mobility that decreases upon interaction with the partner unit, case (B), are provided by the oxidoreductases [PDB:1a05, 1gdh], the hydrolase [PDB:1hii] and the calcium-binding protein [PDB:1a03]. All these complexes have a type I interface. The best examples of fluctuations not appreciably affected by the partner, case (C), are observed for the chemokine [PDB:1a15], the transcription factor [PDB:1a0a], and the leucine zipper [PDB:1a93]. All these complexes are of type II. It is interesting to notice in Figure 4a that, while examples of case (A) are found in complexes of type I, II and III, the behavior (B) is found only in type I, and the behavior (C) is found only in types II and III. Many complexes of type I are examples of behavior (B), with the best-fitting line in the figure having a very small slope. Furthermore, in most dimers with type II interfaces, the partner does not seem to affect the intrinsic fluctuations of the monomer residues (as highlighted in the figure by the interpolating line, with angular coefficient close to 1). By converse, in type III instances it appears that the partner partially influences the amount of fluctuation of the semi-interface residues (as highlighted by the interpolating line, with angular coefficient close to 1/2), although a definite conclusion cannot be drawn in this case due to the limited size of the sample. It can also be observed in Figure 4b that the examples of behavior (B) are obligate complexes, while examples of behavior (C) came from obligate or non-obligate complexes. Furthermore, the size of the interfaces seems to be correlated with the observed behavior. All examples of behavior (B) have an interface area larger than 3000Å^2^, while behavior (C) is observed in interfaces of medium size. Behavior (A) is observed both in large and medium interfaces.

**Figure 5:**
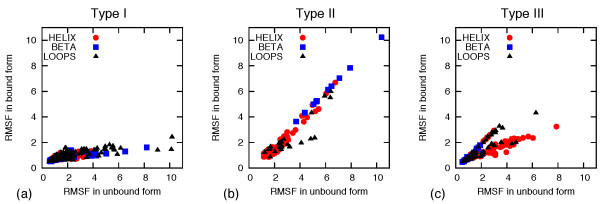
**Scatter plot of the mobility of amino acid at semi-interfaces of bound and unbound monomers**. The symbols and colors of the data points reflect the secondary structure type.

Finally, we consider the secondary-structure content of the semi-interfaces and its correlation with the three types of scenarios. To address this point, the data of Figure 4 have been reproduced in Figure 5 using a color scheme depending on the secondary structure to which each amino acid belongs. The plot for type I, see panel (a), indicates that the highest "unrestricted" fluctuation is for interface residues belonging to loops and beta strands. These amino acids experience the largest variation in the fluctuation due to the influence of the partner monomer. In the plot for type II, see panel (b), the interface residues are mainly in alpha-helices (excluding some beta strands that came from [PDB:1a15]). In the plot for type III, see panel (c), most of the more fluctuating residues are alpha-helices.

## Discussion

The extent to which the mobility of interfacial amino acids changes from the unbound to the bound forms differs across the three main classes of interfaces [35]. For large interfaces of type I the interaction of the partner causes an appreciable loss of mobility compared to the unbound case. However, this intuitive result does not extend to the case of type II interfaces of medium size. For the latter, interface residues remain mobile even in presence of the partner monomer. An attempt to rationalize these observations can be made by considering the interplay between enthalpic and entropic effects on the stability of protein dimers. It is known that entropy plays a fundamental role in the binding processes, and several studies have shown that the entropy associated with the structural fluctuations or vibrations makes a substantial contribution to the association free energy of a complex [63,64]. The precise calculation of this entropy is a challenging task even for non-obligate complexes, and therefore it is beyond the scope of the present study to attempt a quantitative estimate of this contribution to the formation or dissociation of the (often obligate) complexes considered here. Nevertheless, the following considerations can provide a heuristic interpretative framework for the obtained results. A decreased mobility of interface residues in the presence of the monomeric partner expectedly indicates that the partner unit provides a significant limitation to the conformational space of the semi-interface. This suggests that the dimer is likely stabilized by the formation of enthalpically-favorable inter-monomeric interactions. Without such favorable interactions it would become entropically favorable for the monomers to separate as each semi-interface would acquire appreciable structural fluctuations.

This hypothesis is consistent with the different behavior observed for large versus small interfaces. In fact, a large interaction surface can more easily lead to a large enthalpy gain and can compensate for these entropic effects. Instead, the absence of a significant loss of interface mobility between the bound and unbound forms suggests that a smaller enthalpic gain is required to stabilize the dimer. In accord with this and previous observations we notice that dimeric interfaces to which this applies are either small or at most medium-sized and, furthermore, include non-specific interfaces which are more versatile in binding different partners.

Furthermore, if the residues at the semi-interface have an intrinsically-low mobility then binding will not appreciably modify the fluctuation amplitude of the semi-interface. In this case, the entropic change going from the bound to unbound forms would consequently be small and would not need to be compensated by a large enthalpic gain. This would explain why this specific behavior was found across the different interface types, including medium-sized and non-specific ones (i.e. similar interfaces that are observed also in structurally and functionally different complexes).

Analogous considerations would then suggest that this hypothesis should hold for semi-interfaces whose mobility is largely unaffected by the presence of the partner monomer. This behavior could reflect a well-tuned steric and dynamical complementarity of the interacting semi-interfaces. This possibility is reinforced by the fact that this behavior is frequently observed for dimers with type II interfaces, where both semi-interfaces are specific (being conserved among members of the same cluster), and much less frequently for the less specific type III entries, where only one side is conserved.

## Conclusions

A dataset of 22 representative dimeric protein complexes, covering the three known main types of interfaces [29], was characterized to highlight and compare the salient structural and dynamical properties of the dimers.

In particular, an elastic network model was used to characterize the intrinsic fluctuation dynamics of the dimers and their constitutive monomeric units. The analysis of the degree of mobility of the amino acids for the bound monomer indicated that the regions taking part in dimeric semi-interfaces have, on average, a reduced dynamical mobility compared to other regions at the surface, consistently with previous studies [16-19]. This result poses the question of whether the limited mobility reflects an intrinsic property of the semi-interface mobility or if it is due to the presence of the partner semi-interface.

To address this point we proceeded according to a two-tier scheme, where the fluctuation dynamics was computed for unbound monomers, thereby highlighting dynamical features that exclusively depend on the individual monomeric unit, and for the bound forms. By comparing the change in interfacial mobility going from bound to unbound forms, major differences were observed across the three known main types of interfaces. In particular, interface residues of obligate dimers and of complexes with type I interfaces (those where the same interface appears in molecules with the same biological function) display the largest decrease in mobility of the semi-interface regions when the partner monomer is present. On the basis of the obtained results it can be hypothesized that the above-mentioned complexes are stabilized by more favorable enthalpic contributions compared to non-obligate and type II or III instances.

Indeed, for several complexes with type II interfaces (those where the same interface can bind functionally-and structurally-different proteins), the mobility of amino acids at the complex semi-interface is mostly unchanged going from the unbound to bound forms. It is indicative that the chemical and structural complementarity [1,11-15] of the two-semi-interfaces is accompanied by a well-tuned mobility and dynamical complementarity [1,11-15].

The results provide a valuable starting point for a future, more extensive characterization of the interplay of enthalpy and configurational entropy in the stabilization of dimeric complexes in dependence of their size and obligate/non-obligate nature. In addition, the discriminant mobility criteria identified in this study ought to be useful for the *a priori *characterization and classification of novel types of dimeric complexes.

## Methods

### Dataset of protein-protein complexes

The data set of complexes investigated here was obtained starting from that of protein-protein interfaces of Keskin *et al*. [35]. In that work, all the multichain PDB entries in the Protein Data Bank [65] have been considered and used the get all the two-chain combinations. These were extracted; filtered in order to keep only interfaces of a reasonable size; grouped into clusters where members of the same cluster have structurally similar interfaces; filtered again to remove redundancy in sequence among members of a same cluster; and finally the clusters were classified as type I, II or III according to the degree of structural similarity of the members of the cluster. A cluster is classified as type III if its members have only one structurally similar semi-interface. Otherwise the cluster is classified as type I or type II, respectively if its members have or do not have the same global fold. A total of 43, 13 and 47 clusters are classified as type I, II and III respectively. The parental chains of members of the same cluster belong to the same functional family for clusters of type I, and may belong to different functional families for clusters of type II or III [37,38].

For simplicity, the analysis was limited to PDB entries with complete structural information of protein-protein dimers, according to the UNIPROT annotation [66,67], and whose internal dynamics can be adequately modeled using an elastic network model approach (see subsection: "Evaluation of Amino Acids Mobility"). In particular, if the number of zero-energy modes of a dimer, and of each of the constituent monomers, provided by the *β*-Gaussian network model is larger than the six expected for the rigid-body motion, the dimer was excluded form the dataset.

The selection returned 12 dimers of type I (covering 8 different groups); 8 of type II (covering 6 groups) and 9 of type III (covering 8 groups). To limit the redundancy of the dataset, only one representative was retained for each group (the one ranked first in the clustering of Keskin *et al*.). The list of representatives is given in Table 1.

### Protein-protein interaction: obligate, non-obligate and crystal packing

Protein-protein interactions observed in the PDB files may correspond to non-specific crystal packing contacts or biologically relevant interactions. Biologically relevant interactions further divide into non-obligate and obligate, depending on whether the monomers are or are not stable on their own. A recently developed automated probabilistic classification method, NOXclass [68], was used to distinguish between obligate, non-obligate and crystal-packing interactions in the dimers of Table 1. The indicated classification is the one having the highest probability according to NOXclass.

### Protein-protein Interface and Semi-interface

The interface region in protein-protein complexes was defined following ref. [69] which is based on the comparison of the accessible surface area (ASA) of amino acids in the bound and unbound forms. The ASA per residue as well as the total ASA, for each of the studied protein-protein complexes and for the isolated components, were obtained using the program NACCESS [70], with a probe sphere of radius 1.4Å. Since the complexes studied here consist of two chains, the residues at the interface are divided into those which belong to the first or second chain, which constitute the two semi-interfaces of the complex. The interface size is simply given by the number of residues that constitute the interface, and analogously the semi-interface size is the number of residues that constitute the semi-interface.

The interface area is defined, according to [2], as the area of the accessible surface on both partners that becomes inaccessible to the solvent due to the protein contacts. It is calculated as the sum of the ASA of the isolated components minus that of the complex.

### Surface Residues

The residues in a protein can be divided into surface and core residues. According to [69], surface residues are defined as residues having a relative accessible surface area (RASA) greater than 5%. The RASA per residue were obtained using the program NACCESS [70].

### Secondary Structure Evaluation

The secondary structure content of the studied proteins and of their interfaces has been assigned using the DSSP program [71], which defines seven secondary structure states: H (alpha helix), B (residue in isolated beta-bridge), E (extended strand, participates in beta ladder), G (3_10 _helix), I (*π *helix), T (hydrogen bonded turn) and S (bend). We then performed subdivision of the amino acids in terms of helix (H, G, I), strand (B, E) and coil (T, S, or blank space).

### Evaluation of Amino Acids Mobility

#### *β*-Gaussian Network model

Proteins, in thermal equilibrium, experience internal large-scale concerted movements around their native state. These collective movements involve most of the amino acids of the protein, and they are often functionally important since they are strongly related to the conformational changes of the protein. The collective large-scale character of these movements justifies the development and use of simplified approaches to predict and characterize them. A standard way to proceed is using coarse-grained elastic network models [39,41,42,45-49,51,52], which take as input the *C*_*α *_positions of the protein native state, and estimate the energy cost of deviations form the native state by adopting harmonic approximations. It is worth noting that fluctuations predicted in this way will refer only to the backbone motion, not to the side-chain's, since only *C*_*α *_positions are used to calculate the free energy.

Following these approaches, we have adopted here the *β*-Gaussian Network model (*β*GM) [47], which has been validated against a number of atomistic molecular dynamics simulation [47,58-60]. *β*GM uses the following quasi-harmonic approximation of the free energy *F*: a displacement $\delta \text{r}=\{\delta {\vec{r}}_{1},\delta {\vec{r}}_{2},...,\delta {\vec{r}}_{N}\}$ from the native state of the protein (where $\delta {\vec{r}}_{i}$ is the displacement of the *i^th ^C*_α _atom and *N *is the number of amino acids in the protein) is penalized by an increment of free-energy:

(1) $$\Delta F(\delta \text{r}\text{)}=\frac{k}{2}\delta {\text{r}}^{T}M\delta \text{r}$$

where *M *is a 3*N *× 3*N *symmetric matrix which accounts for the pairwise interaction between amino acids. This interaction is assumed to be in the form of a quadratic potential, and it accounts for the chain connectivity and the interaction between amino acids within a distance cut-off *R_c _*= 7.5Å, considering both the position of the *C*_*α *_atoms and the orientation of the side-chain (which is obtained assigning the *C_*β *_*positions from the *C*_*α*_'s). More details are provided in ref. [47]. The model has a single phenomenological parameter:, namely the amplitude of the quadratic potential, *k*, that has been factored out in (1). Collective large-scale movements of the system correspond to the low-energy modes of (1), which are obtained diagonalizing the matrix *M*. For globular proteins, the matrix has six null eigenvalues that correspond to rigid-body rotations and translations of the molecule. For non globular proteins with very exposed loops or termini, extra zero (or nearly zero) eigenvalues are typically observed. This indicates that groups of amino acids experience a diffusive motion that the elastic network approach is not suited to model correctly, so its predictions are not trustable. Clearly, these cases have been eliminated from the dataset. Let us indicate the normalized eigenvectors of *M *as ${\text{v}}^{\alpha }=\{{\vec{v}}_{1}^{\alpha },{\vec{v}}_{2}^{\alpha },...,{\vec{v}}_{N}^{\alpha }\}$ and the corresponding eigenvalues λ_α _(in increasing order for *α *= 1,..., (3*N *- 6), having removed the roto-translations). Eigenvectors associated with the lowest eigenvalues give the directionality of the low-energy motions; the magnitude of the fluctuation along an eigenvector is directly proportional to the inverse of the relative eigenvalue.

Within the model, the thermal fluctuation of the amino acid *i *satisfy the relation:

(2) $$\langle {\left\|\delta {\vec{\text{r}}}_{i}\right\|}^{2}\rangle \propto \sum_{\alpha =1}^{3N-6}\frac{{\left\|{\vec{v}}_{i}^{\alpha }\right\|}^{2}}{\lambda_{\alpha }}$$

where the constant of proportionality depends on the temperature *T *of the system and on the value of the constant of the quadratic potential *k*. It is clear that a precise estimation of these quantities is possible, comparing the predictions with temperature-factors reported in X-rays crystallographic structures or with molecular dynamics simulations. Nevertheless in this work we are mainly interested in changes of mobility, therefore we have fixed *K_B_T *and *k *to 1, so that the estimated fluctuations are expressed in a common unit scale for all the proteins.

This yields the following expression for the root mean square fluctuation (RMSF) of the *i^th ^*amino acid:

(3) $$RMSF(i)=\sqrt{\sum_{\alpha =1}^{3N-6}\frac{{\left\|{\vec{v}}_{i}^{\alpha }\right\|}^{2}}{\lambda_{\alpha }}}$$

which has been used as measure of its degree of mobility.

Finally, let us note that *β*GM, as other elastic network models, is "native-centric", i.e. it models around the input structure and gives predictions in its center of mass.

#### Thermodynamic Integration

The complexes considered in this work are dimers, constituted by two monomers, that we will call here A and B. We want to study the mobility of monomer A in the bound form, i.e. we want to compute the degree of mobility of amino acids of monomer A, in its center of mass, taking into account the presence of B. In order to do that, the amino acids of the complex have to be divided between those of monomer A and of B. The free-energy (1) can be consequently rewritten as follows:

(4) $$\Delta F(\delta {\text{r}}_{A},\delta {\text{r}}_{B})=\frac{k}{2}{\left(\begin{array}{c} \delta {\text{r}}_{A}\\ \delta {\text{r}}_{B} \end{array}\right)}^{T}\left(\begin{array}{cc} M_{A} & G\\ G^{T} & M_{B} \end{array}\right)\left(\begin{array}{c} \delta {\text{r}}_{A}\\ \delta {\text{r}}_{B} \end{array}\right)$$

where *δ***r**_*A *_and *δ***r**_*B *_are the displacements of amino acids in monomer A and B, respectively; *M_A _*and *M_B _*are the interaction matrices for A and B, respectively; *G *and its transpose *G^T ^*contain the coupling between A and B.

By canonically integrating over the degrees of freedom of monomer B, it can be shown [31,72] that the effective free energy for the residues in monomer A, under the influence of monomer B, have still a quadratic form:

(5) $$\tilde{F}(\delta {\text{r}}_{A})=\frac{k}{2}\delta {\text{r}}_{A}^{T}{\tilde{M}}_{A}\delta {\text{r}}_{A}$$

where ${\tilde{M}}_{A}=(M_{A}-GM_{B}{}^{-1}G^{T})$.

The calculation of the degree of mobility of monomer A is therefore obtained diagonalizing the matrix ${\tilde{M}}_{A}$ and using equation (3).

## Authors' contributions

All authors conceived and designed research. AZ carried out the calculations and analyzed results. CM contributed in analyzing data. The manuscript was drafted by AZ and CM and revised by OK and RN. All authors have read and approved the final manuscript.

## Supplementary Material

Additional file 1 **PDF file with additional figures and a table**. Click here for file
