# Supplementary material for: Comparing interfacial dynamics in protein-protein complexes: an elastic network approach
Source: BMC Struct Biol. 2010 Aug 8;10:26. doi: 10.1186/1472-6807-10-26 (PMC2927602; doi:10.1186/1472-6807-10-26)
Supplement: Additional file 1 — PDF file with additional figures and a table. [file 1472-6807-10-26-S1.PDF]

Supplementary Information:
Comparing interfacial dynamics in protein-protein complexes: an elastic network approach

Andrea Zen[1], Cristian Micheletti[1], Ozlem Keskin[2], Ruth Nussinov[3,4]
[1] *SISSA, CNR INFM Democritos and Italian Institute of Technology, Via Bonomea 265, 34136 Trieste, Italy*
[2] *Center for Computational Biology and Bioinformatics and College of Engineering, Koc University, Rumelifeneri Yolu, 34450 Sariyer Istanbul, Turkey*
[3] *Basic Research Program, SAIC-Frederick, Inc., Center for Cancer Research Nanobiology Program, NCI-Frederick, Frederick, MD 21702, U.S.A.*
[4] *Sackler Institute of Molecular Medicine, Department of Human Genetics and Molecular Medicine, Sackler School of Medicine, Tel Aviv University, Tel Aviv 69978, Israel*

RMSF distributions for interface and surface residues

	Interface mean±std.dev.	Surface (not interface) mean±std.dev.	distribution
ALL	1.19±0.95	1.54±2.17	Fig.1
Non Obl.	1.51±1.02	2.09±3.11	Fig.2(a)
Obl.	1.12±0.95	1.47±2.03	Fig.2(b)
Type I	0.85±0.24	1.06±0.37	Fig.3(a)
Type II	2.23±1.71	2.33±4.08	Fig.3(b)
Type III	1.39±0.87	1.79±1.64	Fig.3(c)

TABLE I: Average and standard deviation of the RMSF, in the units of the elastic network model, for residues at the monomeric (bound) semi-interfaces and in non-interfacial surface regions. As indicated in the first column of the table, the statistical quantities are calculated for the first monomer of the full set of 22 representative dimers (first row) or over its various subtypes. The corresponding RMSF probability distributions the are shown in the following figures.

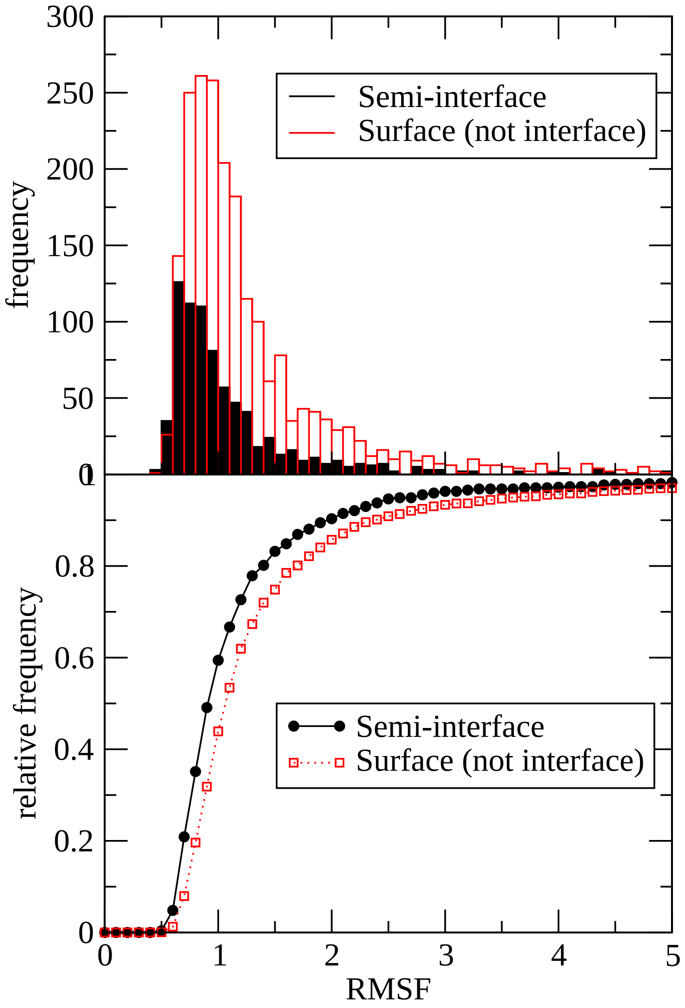

FIG. 1: Top: distribution of the RMSF, in the units of the elastic network model, of residues at monomeric (bound) semi-interfaces and of residues in non-interfacial surface regions of the considered monomers. The associated normalized cumulative distribution is shown in the bottom panel.

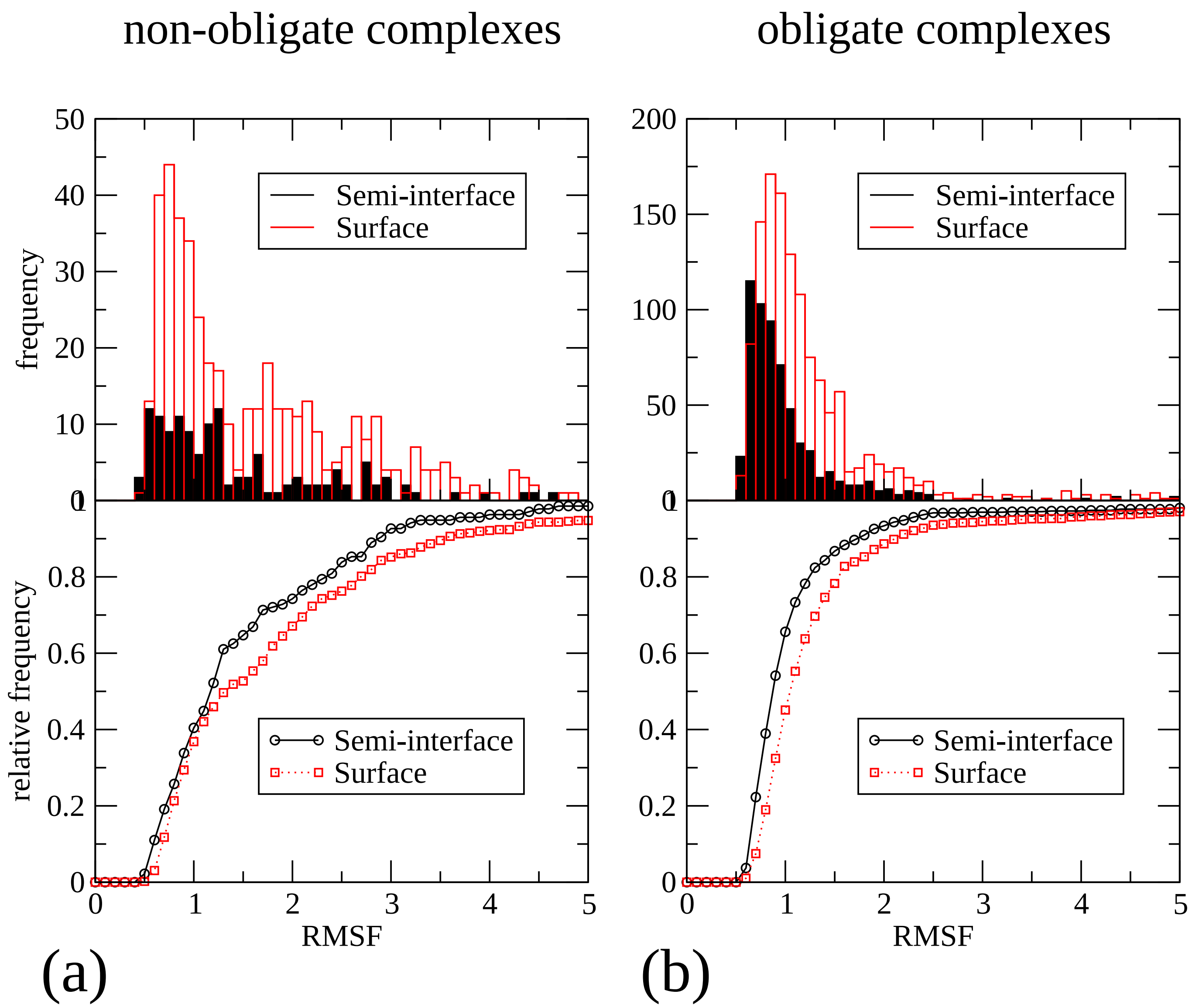

FIG. 2: Same representation of fig. 1. The data are presented separately for obligate and non-obligate complexes.

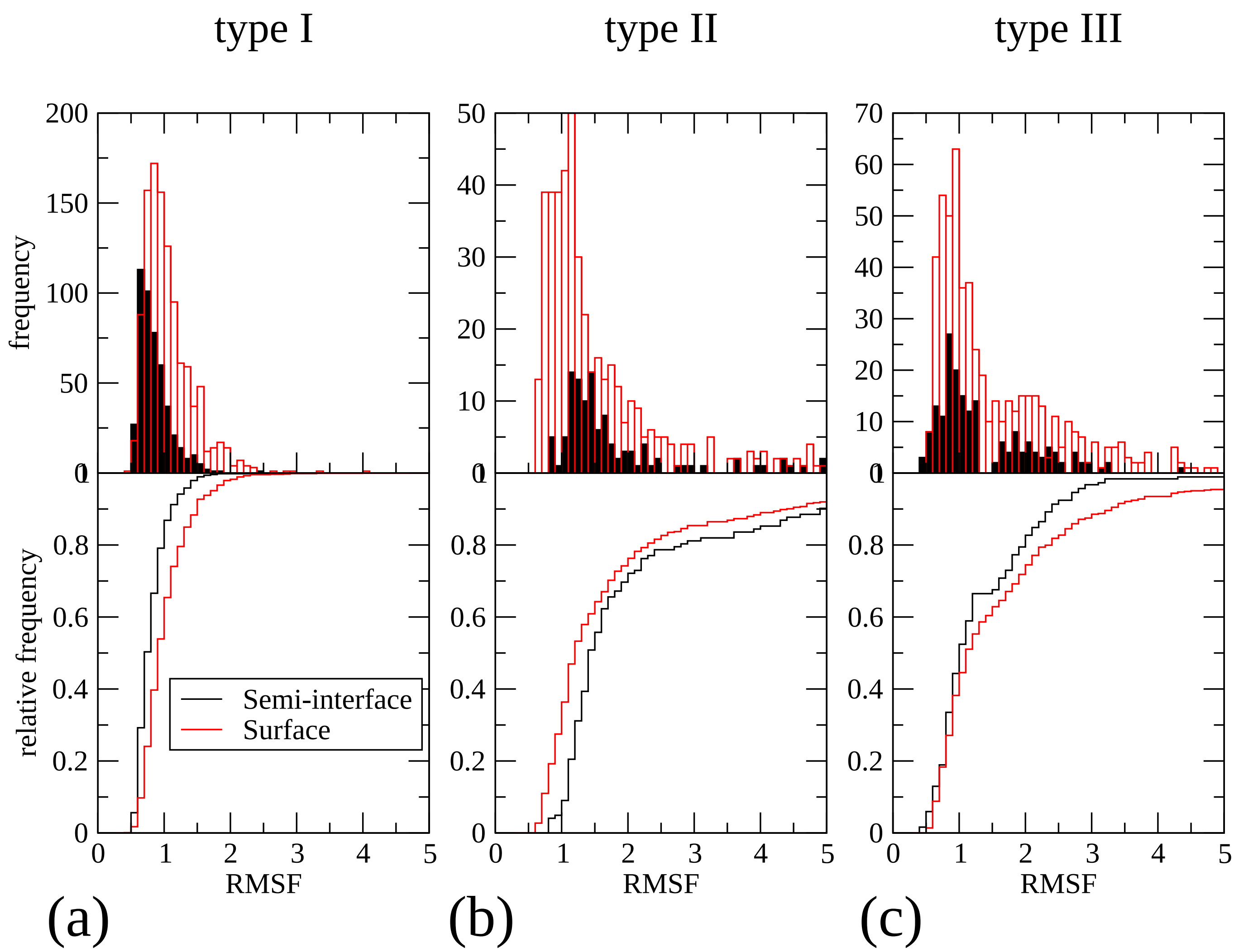

FIG. 3: Same representation of fig. 1. The data are presented separately for the three types of interfaces.

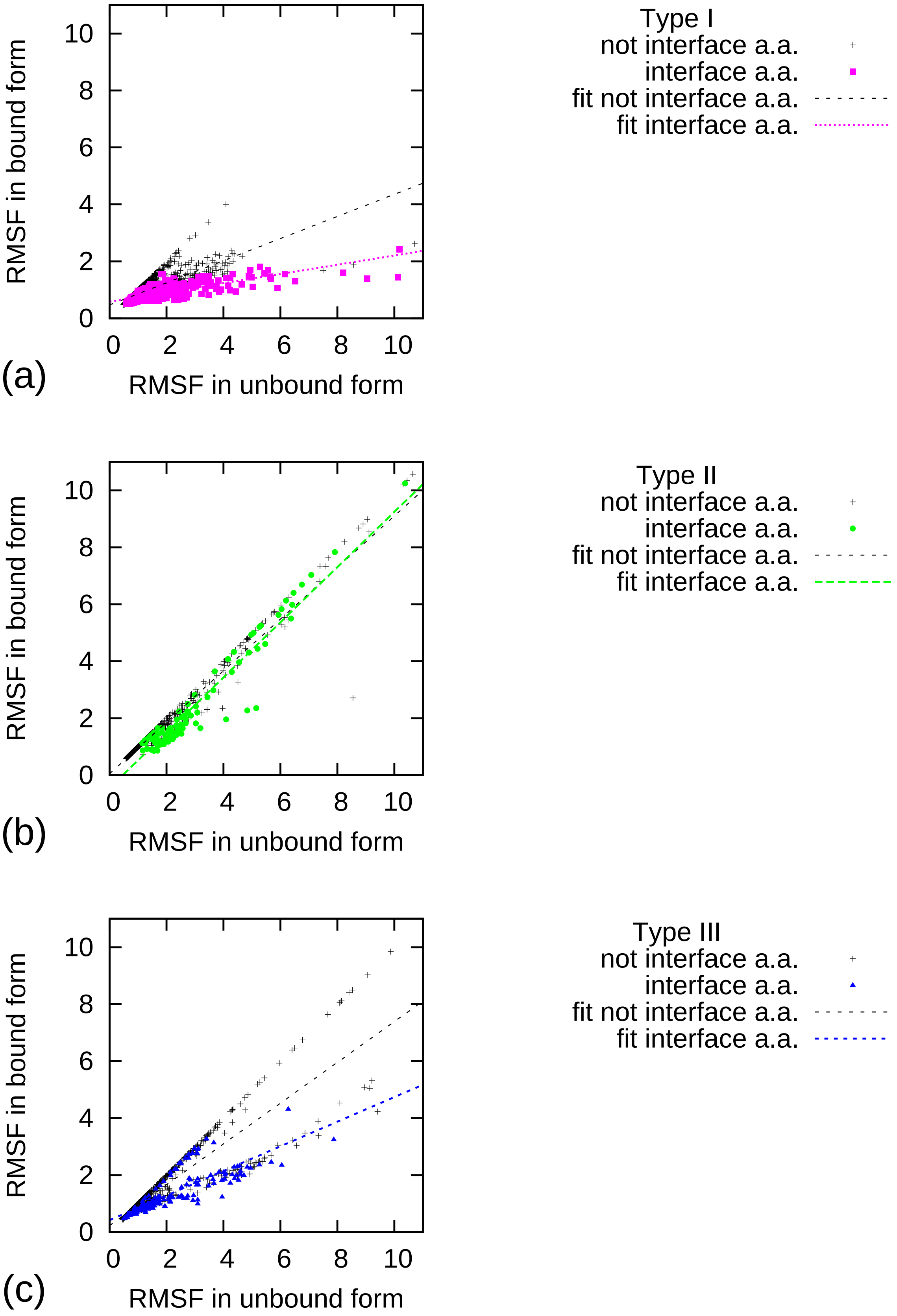

FIG. 4: Scatter plots in panels (a), (b) and (c) show the m Mobility (root mean square fluctuation) of amino acids at the semi-interfaces of *bound* and *unbound* monomers, compared with the mobility of the amino acids that are not at the interface. Panels (a), (b) and (c) pertain to dimers whose interface type is I, II and III, respectively.

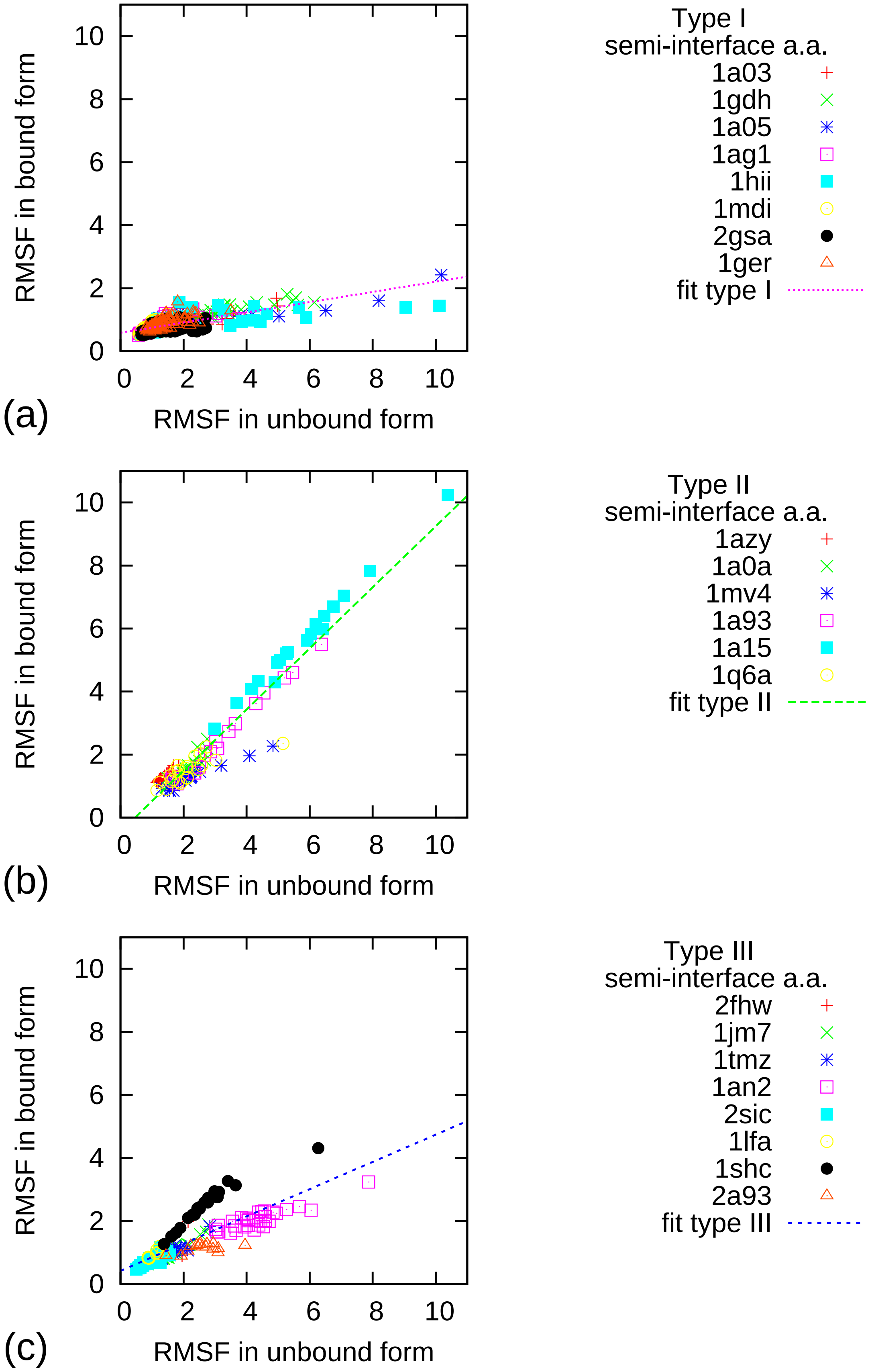

FIG. 5: Scatter plot of the mobility of amino acid at semi-interfaces of *bound* and *unbound* monomers (separated according to interface types).
